# The R.M.O. and the County Court

**Published:** 1908-02-22

**Authors:** 


### Resident Medical Officers' Department.

### THE R.M.O. AND THE COUNTY COURT.

### II.—MEDICAL FEES IN LEGAL CASES.

We come now to the consideration of fees for evidence in compensation cases and other civil actions in County Courts. When a solicitor serves a subpœna he usually pays a sum of money with it, known as "conduct money." If the Court is within a reasonable distance of the hospital at which the R.M.O. resides, this fee as a rule will be one guinea. Before accepting either guinea or subpœna it should be made quite clear that a further fee will be required for giving evidence when the case is called. The conduct money is practically a retainer, and a fee for any expenses incurred in reaching the Court. This is a perfectly fair way of regarding it, and is not a piece of professional avarice; for when once a subpœna has been accepted, the witness must attend at no matter how great inconvenience, and he may find the case postponed or adjourned to some future date, on which his attendance is equally compulsory. It should never be admitted that the conduct fee is part of the payment for giving expert evidence, and it should be definitely explained to the solicitor when the subpœna is tendered what this subsequent fee will be. In a County Court case two guineas, with half that sum for every attendance after the first one, is a very reasonable minimum; more than that can often be quite fairly expected and obtained.

Unless the firm of solicitors be well known as a respectable one, it is necessary to insist on the payment of the agreed sum before being sworn: otherwise the debt may be repudiated, for there is no remedy at law by which it can be enforced. An expert witness can refuse to be sworn if he has not received his fee, and the judge will uphold him, and direct that the money be paid. It requires perhaps a little moral courage to take this course, but even apparently respectable solicitors are not above the very despicable form of meanness described; a firm of considerable standing in the City of London tried it upon the writer once, and it was only by sending them two letters accusing them outright of swindling that they could be induced to pay the debt, which had been twice acknowledged by one of their managing clerks.

There is no objection, as a moment's thought will show, to a medical witness accepting a subpœna and conduct money from each side. Otherwise a litigant whose cause is likely to be damaged by medical testimony might subpœna the witness and then omit to call him. Only one fee for evidence can, however, be claimed, as one side or the other get the opportunity of obtaining the evidence they want by cross-examination.

It frequently happens that an R.M.O. appears as a witness for some patient claiming under the Workmen's Compensation or the Employers' Liabilities Acts, and that the real defendant to the action is one of the insurance corporations which underwrite this sort of risk. Such companies often regularly employ a medical man to examine claims and to give evidence against any that are exaggerated or fraudulent. These gentlemen by long experience are very astute in recognising any bogus claim, and at the same time reasonable about any legitimate one. The R.M.O subpœnaed in such a case will generally find that a preliminary conference will result in an agreement as to the extent of disablement, etc., which is not unjust to either side; he must, of course, be very careful that his patient's interests are not prejudiced in any way by an exacting "company doctor."

It has occasionally been discussed whether such a conference is permissible: without entering into any long argument, it may be said that jurists admit the principle, and that those with practical experience are convinced that it does a very great deal of good and little or no harm.

A further point in connection with insurance medical referees arises sometimes when a company sends one to make a report on an in-patient of a hospital. The official notes of the case taken by the house surgeon are the property of the hospital, and, moreover, they contain the confidences of the patient towards his medical attendant. For both reasons the visiting referee should be rigidly denied access to them. Nor need the resident furnish any information except for a fee. Common courtesy, however, requires that the visitor be allowed to see and speak with the patient, and even make such a cursory examination as is not inconsistent with his state of health. To regard the medical referee as an interloper who is pouching the resident's perquisite is a grasping and jealous attitude unworthy of our profession.

Before we leave this subject of medical reports for solicitors, insurance companies, and the like, it is necessary to be very emphatic on one point. Never in any circumstances should a medical man report about one of his patients, whether in hospital or private practice, without the knowledge and consent of the patient himself, expressed in writing or before witnesses. This one golden rule must never be violated, for the consequences of doing so may be disastrous in the extreme.

